# Metabolic effect of bodyweight whole-body vibration in a 20-min exercise session: A crossover study using verified vibration stimulus

**DOI:** 10.1371/journal.pone.0192046

**Published:** 2018-01-31

**Authors:** Chiara Milanese, Valentina Cavedon, Marco Sandri, Enrico Tam, Francesco Piscitelli, Federico Boschi, Carlo Zancanaro

**Affiliations:** 1 Department of Neurosciences, Biomedicine and Movement Sciences, University of Verona, Verona, Italy; 2 Department of Computer Science, University of Verona, Verona, Italy; University of L'Aquila, ITALY

## Abstract

The ability of whole body vibration (WBV) to increase energy expenditure (EE) has been investigated to some extent in the past using short-term single exercises or sets of single exercises. However, the current practice in WBV training for fitness is based on the execution of multiple exercises during a WBV training session for a period of at least 20 min; nevertheless, very limited and inconsistent data are available on EE during long term WBV training session. This crossover study was designed to demonstrate, in an adequately powered sample of participants, the ability of WBV to increase the metabolic cost of exercise vs. no vibration over the time span of a typical WBV session for fitness (20 min). Twenty-two physically active young males exercised on a vibration platform (three identical sets of six different exercises) using an accelerometer-verified vibration stimulus in both the WBV and no vibration condition. Oxygen consumption was measured with indirect calorimetry and expressed as area under the curve (O_2(AUC)_). Results showed that, in the overall 20-min training session, WBV increased both the O_2(AUC)_ and the estimated EE vs. no vibration by about 22% and 20%, respectively (P<0.001 for both, partial eta squared [η^2^] ≥0.35) as well as the metabolic equivalent of task (+5.5%, P = 0.043; η^2^ = 0.02) and the rate of perceived exertion (+13%, P<0.001; ŋ^2^ = 0.16). Results demonstrated that vibration is able to significantly increase the metabolic cost of exercise in a 20-min WBV training session.

## Introduction

Whole-body vibration (WBV) was introduced in the late 1990s and, over the past decade, WBV exercise has become an increasingly popular training modality especially in the fitness field. A number of studies have been untaken in the last two decades, which have demonstrated interesting results where acute and chronic WBV exercise has been used for improving muscle performance [1–3], bone density [4], balance and proprioception [5] and where long-term exposure to WBV has been shown to be associated with fat body reduction [6–8].

During WBV, the subject stands or exercises on a platform, which vibrates at set frequencies (typically between 15 and 70 Hz) and amplitudes (typically between 1 and 10 mm). WBV is considered a light neuromuscular resistance training method based on automatic body adaptations to repeated, rapid and short intermittent exposure to oscillations from a vibrating platform (review in [9]). Its mechanism of action has been related to reflex muscle activation [10] and muscle twitch potentiation [11] as shown by electromyography studies [12]. Abercromby and colleagues [12], also argued that neuromuscular responses during WBV may be modulated by leg muscle co-contraction as a postural control strategy and/or muscle tuning by the central nervous system. Additionally, WBV may induce a muscle tuning response while the subject attempts to dampen the transmission of vibration [13]. Furthermore, WBV is able to facilitate muscle deoxygenation, thereby improving oxygen delivery to the muscle [14], and to stimulate lipolysis via an acute elevation of lipolytic hormones [15] and growth hormones [16–19]. It has also been shown that exercise-involving WBV is able to attain greater metabolic stimulation and greater energy expenditure (EE) in comparison to the same exercise without WBV [20,21]. Oxygen consumption has long been used to determine EE [22], the association between oxygen consumption and EE being expressed in the well-known Weir equation [23].

A number of papers have reported on WBV-associated oxygen consumption measurements and most previous research showed some ability of WBV to increase oxygen consumption vs. control conditions for a variety of vibration stimuli. These also included various exercise protocols and many different age groups of both sexes. Rittweger et al. [21,24,25] showed that WBV in the side-alt modality is able to acutely increase oxygen consumption of the subject with additional loading and with only the subject's bodyweight. Similar findings have since been reported [21,26–28], and more specifically in young males [29], sedentary males [30], young adult females [31], young adult males and females [32], and elderly participants [33]. Cochrane et al. [34] also reported increased oxygen consumption in both healthy young adults and older people using synchronous vibrations with both static and dynamic movement exercises. Subjects with health issues have also been studied and a WBV-associated increase in oxygen consumption was shown in overweight and obese people [6], spinal cord injury patients [35], and chronic stroke patients [36].

Most of the available literature related to the acute effects of WBV on oxygen consumption focused on short-time WBV exposure sessions (i.e., < 10 min) with single exercises or sets of individual exercises. However, longer WBV exposure sessions (20–30 min of intermittent exercise with a combination of different exercises) are currently preferred for WBV training in fitness centers. This is in agreement with the guidelines established by the American College of Sports Medicine (ACSM) [37] for the management of cardiorespiratory training.

Despite the wide interest elicited by WBV as an exercise modality in the fitness field, few papers examined oxygen consumption during a typical WBV session for fitness [38,39] and what is more, the above studies presented conflicting results, Hazell and Lemon [38] demonstrating a significant increase in oxygen consumption while Gojanovic and Henchoz [39] reporting no differences between synchronous WBV and no vibration.

When investigating the effects of WBV, a number of potentially confounding issues should be addressed. Firstly the lack of characterization of the vibration stimulus by means of an accelerometer, taking instead the settings of the machine as the actual vibration stimulus. It has been shown that the frequency and amplitude generated by a device may vary from the preset values or from the values provided by the manufacturer both across and within platforms [40]. Accordingly, there is a need to evaluate the real parameters of the vibrations produced by a WBV device in a given study setting [41] to interpret the oxygen consumption outcomes in a more correct perspective. Secondly, there is an issue concerning the statistical power of the study, a small sample size challenging the significance of results. Actually, the study group in the work of Hazell and Lemon [38] was composed of only eight healthy males, three of which were replaced during exercise data collection, as the original subjects could not be rescheduled. Similarly, the study carried out by Gojanovic and Henchoz [39] has a limited number of participants (n = 10). Thirdly, variability of covariates within the study groups should be taken into account [38]. Such variability may be limited by adopting a crossover study design.

Therefore, it is clear that the metabolic effects of a typical WBV training session for fitness and the possible extent to which such an effect occurs on healthy and physically active individuals remains matter of debate. It is evidently necessary to bridge this gap in our understanding between the currently used conditions in fitness centers and the supporting research on WBV under laboratory conditions. Considering the above issues, we designed a strictly controlled experiment with a crossover design to extend previous findings by accurately assessing the metabolic cost of a typical WBV training session for fitness in an adequate number of participants and with an accelerometer-measured vibration load. According to our own extensive experience and in agreement with others [42] physically active men are the main users of WBV devices in fitness centers, so the experiment was carried out in young fit men.

## Materials and methods

### Participants and study design

**Fig 1:**
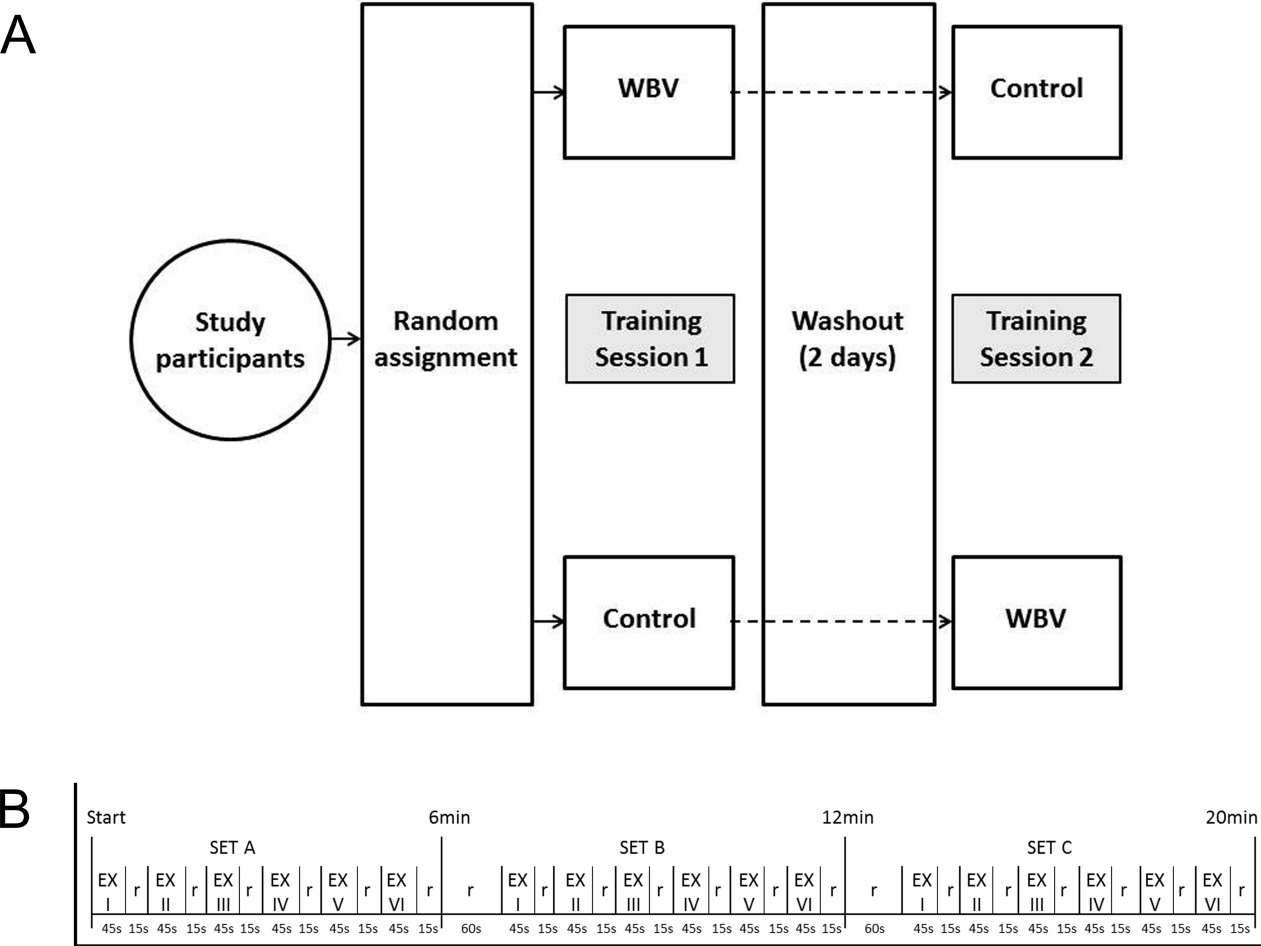
A. block diagram of study design. WBV, whole body vibration. B, schema of the experimental protocol. EX, exercise time; R, between-exercise recovery time.

**Fig 2:**
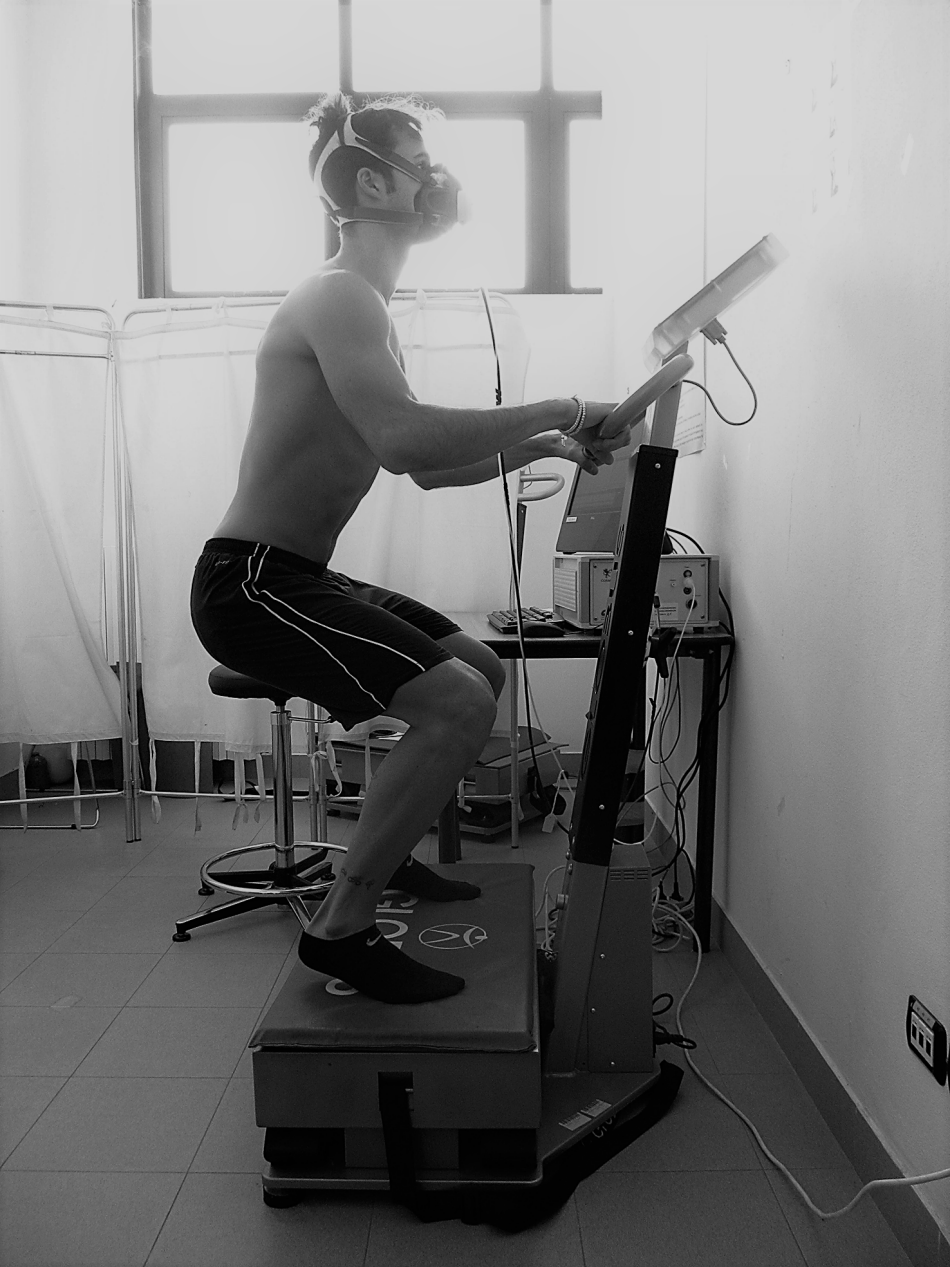
Representative picture of the experimental set up. The participant is performing a squat exercise in the condition currently adopted for healthy subjects in fitness centers.

A total of 28 healthy male Kinesiology students were recruited for this study according to a priori sample size calculations (vide infra), assuming a ~20% drop out. Inclusion criteria were active lifestyle [37] and no WBV experience. All participants passed the PAR-Q health survey [43] before being enrolled in the study. Exclusion criteria were: cardiovascular, neuromuscular, or metabolic conditions that would prohibit exercise; lower-body surgery within the previous six months; use of medications for chronic cardiovascular or neuromuscular conditions; contraindications to WBV according to the manufacturer’s criteria (i.e. diabetes, epilepsy, gallstones, kidney stones, acute inflammations, joint problems, cardiovascular diseases, joint implants, recent thrombosis, back problems such as hernia, tumors, recent operative wounds, or intense migraines). Participants gave their written informed consent. The protocol conformed to the Declaration of Helsinki (revised 2013). Ethical approval for the study was obtained from the Institutional Review Board at the Department of Neurosciences, Biomedicine and Movement Sciences. The crossover design of the study is summarized in the block diagram of Fig 1A. The individual in Fig 2 gave written informed consent (as outlined in PLOS consent form) to publish his image in the paper.

### Characterization of the vibration stimulus

Some studies conducted on various vibrating platforms have highlighted the alterations in vibration parameters when individuals with different body mass perform squatting exercises [40,44–46]. Therefore, a preliminary study was conducted to assess the actual vibration produced by the commercial model of vibration platform (PhysioPlate Fit-Vibe Power, Globus, Codognè, Italy) used in this work. This platform generates a synchronous vertical vibration stimulus whereby the platform oscillates up and down. Using an “unloaded condition” (no load on the platform) and ten different “loaded conditions” (load on the platform ranging up to 112.6 kg) the actual vibration characteristics for each of the five nominal vibration frequencies (i.e, 25, 30, 35, 50, 70 Hz) and two displacement modes (“low” and “high”) implemented in the platform were assessed. Assessment was carried out with a stand-alone 3-axis accelerometer (μ3D Pebble System, ZeroPoint Technology, Johannesburg, South Africa [approximate range: ±100g on 3 perpendicular axes; data acquisition rate: 2000 samples/second/channel; approximate size: 42 x 39 x 12mm]). The accelerometer was solidly affixed to the vibrating plate with double-sided sticky tape at the center of the platform. The accelerometer measurements were stored automatically on a microSD chip inside the accelerometer. Acceleration data were collected over a time span of 5 seconds for each condition. The actual oscillations generated by the WBV device were found almost pure sine waveforms apart the tails due to the start/stop of the platform motion. Oscillations were analyzed using a custom program written in Matlab R2008a (MathWorks, Natick, MA). The frequency of the vibration was evaluated on the basis of the accelerometer read-out i.e., by counting a given number of vibration cycles and dividing that number by the time (in seconds) between the peaks. The resulting ratio was the actual vibration frequency in Hz. The actual values of peak-to-peak displacement, amplitude, peak-to-peak acceleration and peak acceleration were obtained directly from acceleration data as reported by Rauch et al. [41]. Data on the actual vibration frequency, peak acceleration and peak-to-peak displacement in both the “high” and “low” displacement mode in the “unloaded condition” are shown in Table 1. Results highlighted that the actual frequencies in both the “low” and “high” displacement mode were often at variance to the nominal ones. Moreover, data also showed variability in the vibration load according to the load on the platform. For example, at the nominal vibration frequency of 70 Hz and in the “high” displacement mode, the mean error for accelerometer-measured peak acceleration between the unloaded condition and the ten loaded conditions was 5.1%±2.9%. Accordingly, the values of peak acceleration measured in the loading conditions were used to create calibration curves of the vibrational quantities as function of the mass values. By linear interpolation of the nearest values, the vibration quantities were estimated for all the masses in the range 0–112.6 kg with a 0.1 kg step size. Thus, for each individual participant’s mass the vibrational quantities are easily derived from the calibration curves.

**Table 1:** Unloaded configuration for actual vibration characteristics (frequency, peak-to-peak displacement and peak acceleration) measured by accelerometer.

Nominal f (Hz)	Actual f (Hz)		Actual D (mm)		Actual a_Peak_ (g)	
	Low	High	Low	High	Low	High
25 Hz	25.0	23.0	1.3	2.5	1.6	2.7
30 Hz	26.3	29.9	1.3	1.8	1.8	3.2
35 Hz	32.3	30.0	1.0	1.8	2.1	3.3
50 Hz	45.5	43.5	0.9	1.8	3.7	6.8
70 Hz	54.1	58.8	1.0	1.6	5.9	11.1

f, frequency; D, peak-to-peak displacement; Low/High, low amplitude and high amplitude setting of the vibration platform; a_Peak_, peak acceleration.

### Whole-body vibration training session

To date, the optimal combination of frequency and amplitude to be used in intermittent WBV training protocols involving static and dynamic exercises for increasing oxygen consumption has not been clearly determined. However, it has been reported that the increase in oxygen consumption can be parametrically controlled by frequency, amplitude, and the external load applied during WBV [25]. Recently, Kang and colleagues [47] investigated the metabolic responses during WBV combined with body weight squats, pointing out that the greatest increase in oxygen consumption was observed at the maximum frequency tested (50 Hz) and at high amplitude. Accordingly, in order to maximize the potential for detecting differences in oxygen consumption, the platform settings “High” and “70Hz” corresponding to an actual peak-to-peak displacement of 1.9 mm and vibration frequency of 55.44 Hz (with a mass of 70 kg on the platform) were selected to deliver the vibration stimulus in this work.

This study adopted a randomized 2-sequence (AB | BA), 2-treatment (WBV vs. no vibration) crossover design. At the beginning of the study, the 28 study participants were randomly assigned to one of the two sequences using the Wei’s urn design, a randomization technique for balancing treatment assignments [48]. Participants randomized to the AB sequence performed a training session with WBV and after a washout period of 48h, a training session with no vibration. Participants randomized to the BA sequence performed a training session with no vibration and after 48h a training session with WBV. A 48h washout period has been shown to be enough for complete recovery from a period of moderate to heavy exercise from a functional/metabolic point of view [49]. Similarly, de Hoyo et al. [50] reported that a washout period of 48h after a six-minute bout of WBV is enough for plasma markers of muscle damage to return to baseline in young, healthy, physically active participants.

Participants were asked to refrain from vigorous exercise for at least 12h before they arrived at the laboratory. The testing protocol was explained to participants and they were given the opportunity to familiarize themselves with the vibrating platform and with the correct rhythm and technique of exercises before the sessions.

Each training session was organized into 3 identical sets (A, B, C) encompassing 6 dynamic exercises each (vide infra). The order of exercises in each set was held constant in AB and BA sequences. A total of 72 repeated measures were taken for each participant: (6 exercises + 6 between-exercise rests) x 3 sets x 2 treatments.

The WBV training session consisted of a 20-min exercise session on the vibration platform with the vibration turned on, while the no-vibration training session was performed on the same platform with the vibration turned off. Following a standardized warm-up, consisting of static and dynamic exercises and stretches, participants completed three sets (A, B, C) of six dynamic exercises (I-VI), involving the major muscle groups of both the upper and lower body. The training session was comprised of (I) squats, (II) push-ups with the hands on the platform, (III) isometric squats, (IV) plié squats, (V) lunges with the lead foot on the platform, and (VI) squats on tiptoes; the protocol (type, duration, and sequence of exercises) was borrowed from a previous work [39]; it represents an intermittent exercise protocol consisting of a balanced mix of exercises closely matching the most currently used conditions of WBV exercise in the fitness field. Blocks placed in front of the vibration platform were utilized for push-ups and lunges so that the hands and feet that were off the platform remained at the same height as the vibrating platform. During the sessions, participants wore cotton socks to avoid any between-participant variance in damping [51]. The rhythm, the range of motion and the body mechanics, that is, the position of the feet on the platform and the position of the spine, arms, legs and head of each subject were continuously supervised by an operator and corrected where necessary. The exercise to recovery ratio was 3:1 (i.e., 45s exercise: 15s recovery); at the end of each set an additional 60s recovery time was allowed. Over the 20-min protocol, the total exercise time was 13.5min and the total recovery time was 6.5min (Fig 1B). Each exercise included 15 repetitions over 45s, each one consisting of a 1s eccentric contraction, a 1s isometric contraction and a 1s concentric contraction, paced by an auditory metronome. The isometric squats were performed over 45s. The heart rate was registered throughout. At the end of each training session, participants were asked to report their rating of perceived exertion (RPE) using the Borg 6–20 scale [52]. The study was completed in winter and spring (December-April).

### Anthropometry and body composition analysis

Body mass (Tanita BWB-800 scale, MA, USA) and stature (Holtain stadiometer; Holtain Ltd., Crymych, Pembs, UK) were measured in all participants to the nearest 0.1kg and 0.01m respectively, with the subject wearing underwear and no shoes. Body mass index (BMI) was calculated as weight (kg)/stature^2^ (m).

### Indirect calorimetry

All measurements were made using an online breath-by-breath analysis of oxygen consumption and carbon dioxide production (Quark CPET; Cosmed, Rome, Italy). The system was calibrated before each test by means of a certified gas mixture (FO_2_: 16%; FCO_2_: 5%, N_2_ for balance) and a 3L-calibrated syringe (Hans Rudolph, Kansas City, MI, USA) according to the manufacturer’s recommendations. Before each exercise session (WBV, no vibration) participants were instrumented, and oxygen consumption was monitored for three minutes at rest in a standing position (basal oxygen consumption) and thereafter along the entire training session. A representative picture of the experimental setup is presented in Fig 2. Among others, the Quark CPET output yields values of oxygen consumption, estimates of EE (Kcal) according to the Weir equation [22] and the metabolic equivalent of task (MET). MET is a practical procedure for expressing the energy cost of physical activities as a multiple of the resting metabolic rate. Because of the intermittent nature of the exercise, no steady-state oxygen consumption was achieved; in this condition, the overall oxygen uptake (mL) better represents the body’s metabolic demand instead of relative oxygen consumption (mL/kg/min). Accordingly, oxygen consumption was calculated as the area under the curve (O_2(AUC)_) using linear interpolation with the composite trapezoid rule [53]. The mean value of O_2(AUC)_, EE, and MET in the participant group were calculated in the WBV and no vibration condition for the following time intervals: whole session (20 min, including exercise and recovery phases), set A, B, and C (6min each, including exercise and recovery phases), total exercise time (13.5min), and total recovery time (6.5min).

### Statistical analysis

Considering the crossover design of the present study, the required sample was estimated a priori [54] using the formula: $n={\left(z_{\alpha /2}+z_{\beta }\right)}^{2}\sigma_{m}^{2}/(2\epsilon^{2})$, where ϵ is the mean value of the differences between the levels of O_2_(AUC) for exercising subjects with and without WBV and σ_m_ was the standard deviation of these differences. Preliminary investigation showed that σ_m_ was approximately 2,300 mL in each experimental group. Setting the minimum relevant clinical difference at 1000 mL (effect size = |ϵ|/σ_m_ ≈ 0.43), the type I error at α = 0.05, and the power 1-β at 0.80, the minimum required total sample size was 21 subjects.

Numerical variables were summarized using mean ± standard deviation (SD) and normal distribution of the data was tested using the Shapiro-Wilk test. Differences between mean values of outcomes in the WBV and no vibration training sessions were analyzed using mixed effects linear models [55], a class of statistical models suitable for analyzing data from studies with longitudinal and crossover design and to model the correlation between the multiple measurements within each individual [56]. The estimated differences in outcomes between treatments (WBV vs. no vibration) were adjusted for individual peak acceleration and baseline oxygen consumption, and exercise set (A vs. B vs. C) by adding these variables to the set of covariates of the regression model. The presence of an effect associated to the order of intervention was investigated when analyzing data with mixed-effects models: a binary “sequence” variable (WBV/no vibration vs no vibration/WBV) was considered and its statistical significance was calculated. The effect size of WBV was estimated using partial eta squared (η^2^, the proportion of variance in the outcome explained by WBV). Following Cohen [57], we interpret estimated η^2^ values as follows: 0.01 small, 0.06 medium, 0.14 large.

Two-tailed statistical tests were used throughout and tests were rejected when P ≤ 0.05. Sample size was calculated using G*Power ver.3.1.9.2 [58]. Statistical analysis was carried out using STATA v.13.1 (Stata Corporation, College Station, TX) and R v.3.2.5 (R Foundation for Statistical Computing, Vienna).

## Results

**Fig 3:**
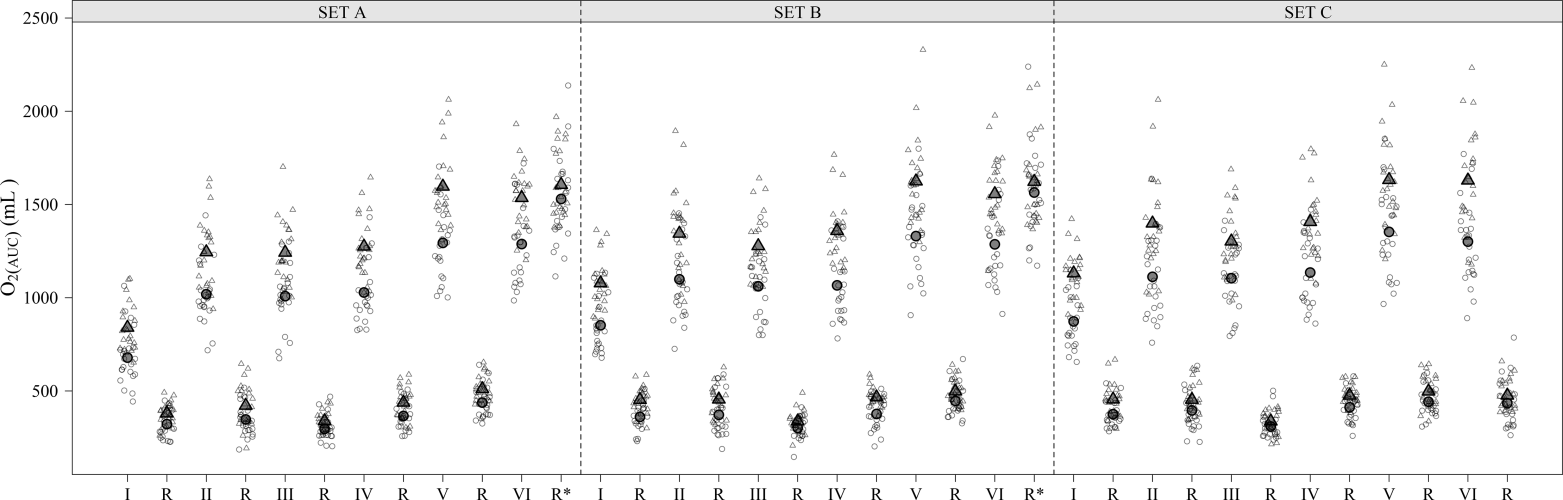
Individual pattern of O_2(AUC)_ in the 22 participants over the whole training session (Sets A-C) in the presence (open triangle) and absence (open circles) of vibration (~56Hz, ~11g). Mean value is indicated by filled symbols. I-VI, sequential exercises (see text); R, between-exercise recovery time. R*, sum of between-exercise and between-set recovery time. Data are adjusted by individual peak acceleration and order of treatment administration (WBV/no vibration vs. no vibration/WBV).

The 20 min WBV training was well tolerated by all participants insofar none of them complained for headache, nausea, vomit, vertigo, or dizziness during or immediately after completing the protocol. None of the participants reported any carryover effects that may have resulted from the previous testing session that required postponement of testing. Six participants did not complete the evaluations and dropped out of the protocol. Four of them missed the second training session for personal reasons; two participants got ill between the first and second training session. Accordingly, a complete set of measurements was available for 22 subjects (age, 26.1±6.11y; body mass, 75.1±8.11kg; stature, 177.6±6.10cm; BMI, 23.8±2.40kg/m^2^), which made the study sufficiently powered. Mean basal heart rate was 72.1±7.34 bpm. The mean characteristics of the vibration stimulus were as follows: peak-to-peak displacement 1.80±0.19mm (amplitude 0.90±0.10mm) and vibration frequency 56.4±1.98Hz corresponding to peak acceleration 11.4±0.49g. Mean heart rate was 146.9±11.73 and 150.3±13.05 bpm during the training session in the no vibration and WBV condition, respectively; the two means were not significantly different (P = 0.368). The time-course of O_2(AUC)_ for the twenty-two participants over the entire WBV and no vibration training session is presented in Fig 3. No significant carry over effect on the outcome mean levels was found in the WBV and no vibration conditions as far as the order of treatment is concerned.

### Effect of WBV vs. exercise alone on the metabolic cost of the entire training session

The values of O_2(AUC)_, EE, MET, and RPE during the entire training session in the presence and absence of WBV are reported in Table 2 together with the respective P value and η^2^. WBV was able to significantly increase O_2(AUC)_, EE, MET, and RPE by ~22%, ~20%, ~5%, and ~12%, respectively.

**Table 2:** WBV-associated changes in several outcome measurements in a group (n = 22) of exercising male participants. Data are mean±SD.

Variable	Training period	No vibration	WBV	P value	η^2^
O_2(AUC)_ (mL)	Entire training session	25453.8 **±** 3890.5	30996.8 **±** 3606.5	<0.001	0.42
	Set A	8085.8 **±** 1211.9	9824.7 **±** 1064.5	<0.001	0.44
	Set B	8554.0 **±** 1357.3	10450.8 **±** 1210.9	<0.001	0.42
	Set C	8814.0 **±** 1382.4	10721.3 **±** 1424.3	<0.001	0.37
EE (Kcal)	Entire training session	144.4 **±** 21.8	173.9 **±** 19.6	<0.001	0.35
MET	Entire training session	5.4 **±** 0.9	5.7 **±** 0.9	0.043	0.02
RPE	Entire training session	12.3 **±** 2.03	13.9 **±** 1.90	<0.001	0.16

O_2(AUC)_, oxygen consumption (area under the curve); EE, energy expenditure; MET, metabolic equivalent of task; WBV, whole-body vibration; RPE, rate of perceived exertion; η^2^, effect size.

**Fig 4:**
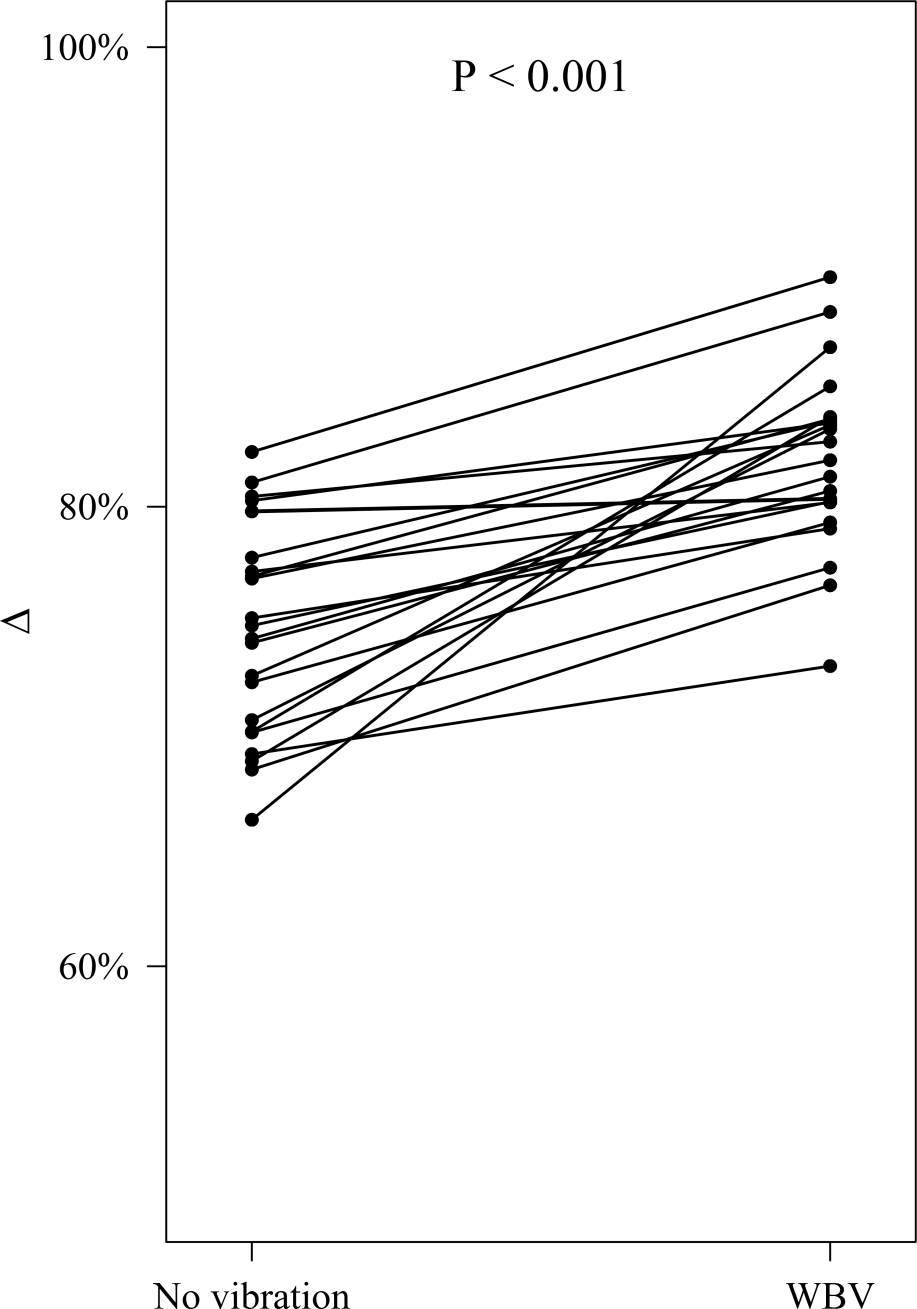
Individual percent reduction (Δ) of O_2_ consumption in the 22 participants during total recovery time (6.5min) along a 20-min training session in the presence (WBV) or absence (no vibration) of whole body vibration. Δ was significantly higher in the presence of WBV. Data are adjusted by peak acceleration and order of treatment administration (WBV/no vibration vs. no vibration/WBV).

When O_2(AUC)_ in the total exercise time (13.5min) of the training session was considered, higher values were found during vibration vs. no vibration (WBV: 24476.6±2881.6mL, no vibration: 19890.8±3026.1mL; P<0.001; η^2^ = 0.44. The order of intervention (WBV/no vibration vs. no vibration/WBV) showed no effect on oxygen consumption (O_2(AUC) =_ 22043.3±4153.5mL and 22386.6±3119.8mL, respectively; P = 0.763). During the total recovery time of the training session (6.5min) O_2(AUC)_ was higher during vibration (WBV:10224.7±1166.9mL, no vibration: 9091.2±1447.4mL; P<0.001; η^2^ = 0.19) as well. Oxygen consumption was higher during the exercise than recovery time in both WBV and no vibration (Fig 3); however, the percentage decrease in O_2(AUC)_ between the total exercise time (13.5 min) and the total recovery period (6.5min) was significantly higher in the WBV vs. no vibration condition (-82.1±3.9% vs. -74.6±4.6%; P<0.001; η^2^ = 0.46) (Fig 4).

### Effect of WBV vs. exercise alone on the metabolic cost of individual set of exercises in a training session

The values of O_2(AUC)_ for each set of exercises (Set A, B, C) in the presence and absence of WBV are reported in Table 2 together with the respective P value and η^2^. WBV was able to significantly increase O_2(AUC)_ by ~21%, ~22%, and ~22% in set A, B, and C, respectively.

When the Quark CPET output was expressed in mL/kg/min and the resulting values analysed the same way as O_2(AUC)_ data, superimposable results were obtained.

## Discussion

This crossover study was designed to test the hypothesis that WBV is able to impose additional metabolic cost to exercise alone in a 20-min WBV training session for fitness. Twenty-two young, physically active males exercised in a 20-min training session that comprised three sets of six different exercises in the presence and absence of an accurately verified vibration stimulus. Results showed that WBV significantly increases O_2(AUC)_ and EE during the 20-min training session. It should be noted that in this work the addition of vibration to exercise was associated with large η^2^ in O_2(AUC)_ and EE changes, indicating that the experiment was able to detect even small changes in the metabolic cost of exercise.

### Energy expenditure increases during whole-body vibration training.

The main finding of this study was that WBV (~56Hz, ~11g) applied in a 20-min training session elicits significantly greater (~22%, P<0.001) oxygen consumption in comparison with exercising at no vibration (Table 2).

The vibration stimulus administered to participants was quantified in terms of frequency and acceleration using an accelerometer taking into account the individual participant’s body mass.

Due to its crossover design, this study minimized variance for the estimated treatment mean difference. Accordingly, data obtained under our experimental conditions strongly support the concept that WBV per se is able to increase oxygen consumption in association with exercise. Under the adopted experimental conditions, a significant increase in oxygen consumption was found during the overall period of exercise (i.e., 13.5 min) as well as recovery (i.e., 6.5 min), demonstrating that the WBV effect continues shortly after the completion of the exercise. The finding of WBV-associated increase in oxygen consumption is supported by the significantly higher (~12%, P<0.001) mean RPE reported by participants after WBV training (“somewhat hard” on the Borg scale) vs. no vibration (“light”). The finding that a higher metabolic cost results in an increased RPE is consistent with previous research [59,60] showing that RPE correlates well with physiological measures of exercise intensity (e.g., hearth rate, oxygen consumption, blood lactate concentration, and respiratory rate both within a person and across workloads and work types. Interestingly, WBV did not associate with significant heart rate changes vs. no vibration in our sample, which deserves further investigation.

Using an accelerometer-verified vibration stimulus for the duration of a typical WBV session for fitness (20 min) our results confirm and expand on previous work on WBV-associated increase in oxygen consumption using mostly uncontrolled vibration conditions and/or shorter periods of exercise [6,24–35,39,61]. Our findings are indirectly supported by recent work using accelerometer-validated vibration stimulus showing that WBV exposure is able to increase oxygen consumption in the absence of associated voluntary exercise in both young adults [62] and chronic stroke patients [36]. In the study from which the WBV training protocol was taken for this experiment [39], no significant difference in oxygen consumption was found during a 20-min WBV session (synchronous vibration) vs. no vibration in association with no significant difference in RPE. There is no obvious reason for such a discrepancy. Possible explanations are the higher vibration frequency adopted in our study compared to that used by Gojanovic and Henchoz [39] (~56Hz vs. 35Hz) and the fact that participants were physically fit instead than sedentary. In fact, it has been shown that metabolic response to WBV may differs according to fitness level and vibration frequency [30].

A statistically significant WBV-associated increase in total EE took place during a 20-min WBV training session (~30 Kcal, ~20%; P<0.001) corresponding to a significant increase of ~0.3 METs (~5%, P = 0.042) (Table 2). These findings are comparable to that of Rittweger et al. [24] and are consistent with data from others. For example, Cochrane et al. [34] showed that a 4min WBV protocol produces an increase of about 0.3 METs versus a no vibration condition. Using a 30-min WBV protocol (exercise:recovery ratio 1:1) oxygen consumption increased by 10% vs. no vibration and by 25% vs. no-exercise on a daily basis [38]. It is interesting to note that previous studies investigating the long-term effects of WBV showed that it is able to induce fat loss in normal weight females [7] as well as overweight and obese people both when used alone [8] or in combination with caloric restriction [6]. These data suggest that the increase in EE associated with regular WBV training would assist weight management given that a significant inverse cross-sectional relationship between activity EE and the percent body fat has been demonstrated in males [63]. In our study, the amount of additional calories burned in the 20-min WBV training session was relatively small and the source of these calories (fat, glycogen, etc.) was not determined; moreover, oxygen consumption was not measured following the training session, thereby missing any post-training effect of WBV. Accordingly, more complete prospective studies are required to assess possible chronic effects of our WBV training protocol on body composition.

In the current study, the WBV training session was split into three sets of consecutive, identical exercises. The WBV-associated increase in oxygen consumption was about ~21%, ~22%, and ~22% in set A, B, and C, respectively, all these figures being statistically significant (Table 2). This indicates that the ability of vibration to impose additional oxygen uptake during exercise is maintained all along the training session suggesting that further set(s) could be added to the protocol in order to enhance total EE, provided that they are well tolerated.

O_2 (AUC)_ was significantly higher in WBV vs. no vibration during both exercise (~23%, P<0.001) and recovery time (~16%, P<0.001). Interestingly, however, O_2_ requests during the overall recovery time decreased to a significantly higher extent in the presence of WBV (~ 82%) vs. no vibration (~74%) (Fig 4). It may be hypothesized that WBV facilitates the physiological mechanisms involved in the rapid decrease in O_2_ consumption after stopping exercise, in possible association with improved neuro-muscle activation and perfusion [29,64–66]. This hypothesis is consistent with findings showing amelioration of muscle performance with WBV [9,67,68] as well as the existence of a positive correlation between an increase in muscle blood flow and performance recovery between bouts of high-intensity exercise [69]. Future work measuring local muscle activation and oxygenation along a 20-min long WBV training session will better characterize this effect of vibration.

### Limitations

This study has some limitations that should be mentioned. Firstly, only one frequency and amplitude were used in a single training paradigm; therefore, the results cannot be extrapolated to different vibration stimuli and/or training paradigms. Previous findings in acute studies [20,34,47] showed a dose-response relationship between the vibration stimulus and oxygen consumption in association with e.g. different degrees of muscle activation. Further study is required to verify how modulation of the vibration stimulus affects the WBV-associated increase in oxygen consumption during longer training. A further limitation is that the actual dose of vibration delivered to participants was not measured by attaching accelerometers to them. Finally, the current findings cannot be generalized to populations other than young, physically fit males.

## Conclusion

This crossover study, carried out under accelerometer-verified vibration conditions, showed that during a 20-min WBV training session vibration significantly increases the metabolic cost of exercise. Due to its ease of administration indoor and outdoor and low cost WBV can be a practical complement in physical activity programs in several settings.

## Supporting information

S1 File Dataset. (PDF)

S2 File Dataset description. (PDF)
