# Supplementary material for: Metabolic effect of bodyweight whole-body vibration in a 20-min exercise session: A crossover study using verified vibration stimulus
Source: PLoS One. 2018 Jan 31;13(1):e0192046. doi: 10.1371/journal.pone.0192046 (PMC5792008; doi:10.1371/journal.pone.0192046)
Supplement: S1 File — (PDF) [file pone.0192046.s001.pdf]

| Id | Age | Sequence | PeakAccel | BodyMass | Height | BMI | HR_bas | HR_V | HR_nV | EE_V |
|---|---|---|---|---|---|---|---|---|---|---|
| 1 | 30 | V_noV | 24,13 | 71,60 | 171,50 | 24,34 | 78,20 | 155,34 | 148,13 | 166,28 |
| 2 | 21 | V_noV | 21,57 | 85,00 | 178,50 | 26,68 | 75,69 | 153,52 | 151,45 | 210,05 |
| 3 | 19 | V_noV | 24,26 | 72,30 | 179,20 | 22,51 | 62,20 | 135,11 | 130,05 | 189,56 |
| 4 | 35 | V_noV | 22,59 | 79,90 | 180,30 | 24,58 | 72,50 | 156,39 | 155,41 | 171,26 |
| 5 | 19 | V_noV | 23,04 | 65,90 | 172,60 | 22,12 | 70,11 | 143,22 | 145,14 | 171,73 |
| 6 | 19 | V_noV | 22,01 | 82,00 | 185,20 | 23,91 | 60,41 | 129,77 | 131,62 | 189,12 |
| 7 | 21 | V_noV | 24,15 | 71,70 | 181,50 | 21,77 | 82,24 | 170,84 | 162,87 | 178,26 |
| 8 | 19 | noV_V | 21,18 | 91,50 | 178,40 | 28,75 | 75,10 | 153,97 | 150,08 | 206,38 |
| 9 | 25 | V_noV | 22,67 | 64,20 | 168,70 | 22,56 | 71,33 | 150,04 | 145,03 | 169,25 |
| 10 | 33 | V_noV | 22,98 | 65,60 | 176,20 | 21,13 | 68,07 | 148,31 | 144,30 | 174,37 |
| 11 | 19 | V_noV | 22,70 | 79,50 | 182,50 | 23,87 | 65,31 | 148,10 | 151,80 | 185,69 |
| 12 | 32 | noV_V | 23,11 | 66,20 | 181,60 | 20,07 | 62,95 | 157,23 | 155,64 | 160,47 |
| 13 | 25 | V_noV | 23,35 | 77,10 | 186,50 | 22,17 | 81,04 | 158,48 | 150,95 | 208,30 |
| 14 | 34 | V_noV | 23,37 | 67,40 | 173,00 | 22,52 | 77,81 | 151,72 | 150,08 | 159,03 |
| 15 | 21 | noV_V | 21,64 | 84,00 | 174,00 | 27,74 | 79,60 | 168,01 | 159,47 | 188,04 |
| 16 | 25 | noV_V | 22,39 | 62,90 | 165,00 | 23,10 | 61,87 | 115,39 | 120,94 | 144,43 |
| 17 | 21 | V_noV | 22,94 | 78,60 | 166,40 | 28,39 | 64,03 | 139,05 | 131,06 | 149,79 |
| 18 | 31 | noV_V | 23,90 | 75,10 | 185,50 | 21,82 | 66,40 | 137,57 | 128,21 | 157,09 |
| 19 | 26 | noV_V | 21,62 | 84,20 | 184,00 | 24,87 | 83,88 | 161,71 | 154,16 | 184,66 |
| 20 | 32 | noV_V | 21,60 | 84,60 | 180,40 | 26,00 | 79,64 | 166,17 | 165,44 | 145,65 |
| 21 | 33 | noV_V | 24,42 | 73,20 | 180,00 | 22,59 | 76,29 | 150,11 | 153,38 | 162,84 |
| 22 | 35 | noV_V | 23,64 | 68,90 | 176,90 | 22,02 | 72,09 | 156,18 | 147,10 | 154,45 |

| EE_nV | METs_V | METs_nV | RPE_V | RPE_nV | AUC_V | AUC_nV | AUC_A_V | AUC_A_nV | AUC_B_V |
|---|---|---|---|---|---|---|---|---|---|
| 129,07 | 5,72 | 5,00 | 16 | 12 | 29218,84 | 21870,07 | 9071,00 | 6897,10 | 10052,26 |
| 187,45 | 6,25 | 6,41 | 17 | 14 | 37277,83 | 32749,77 | 11274,33 | 10434,20 | 12717,39 |
| 169,82 | 6,60 | 6,91 | 14 | 12 | 34432,67 | 31155,53 | 11257,78 | 10490,61 | 11304,62 |
| 158,14 | 5,27 | 5,60 | 14 | 13 | 30073,87 | 27414,54 | 9025,83 | 8355,47 | 10219,56 |
| 131,81 | 6,71 | 5,94 | 10 | 12 | 31056,38 | 23736,27 | 10280,44 | 7640,23 | 10377,49 |
| 151,46 | 2,59 | 2,37 | 13 | 10 | 33382,59 | 26562,31 | 10041,56 | 8178,35 | 11213,65 |
| 134,86 | 6,24 | 5,53 | 14 | 10 | 31383,64 | 23856,69 | 9455,70 | 7581,38 | 10768,00 |
| 170,30 | 5,83 | 5,51 | 15 | 10 | 37489,30 | 30243,94 | 11645,03 | 9227,54 | 12640,13 |
| 157,71 | 5,72 | 6,15 | 13 | 10 | 25834,72 | 20294,91 | 8464,28 | 6519,99 | 8723,96 |
| 139,80 | 6,12 | 5,69 | 10 | 8 | 29347,76 | 21351,39 | 9109,27 | 6852,92 | 9964,81 |
| 125,53 | 5,84 | 4,53 | 13 | 10 | 33341,23 | 22169,17 | 10234,59 | 7097,26 | 11539,98 |
| 118,03 | 5,89 | 4,91 | 15 | 15 | 28670,53 | 20685,00 | 8930,11 | 6381,41 | 9627,98 |
| 169,21 | 6,43 | 6,09 | 13 | 12 | 37657,96 | 29990,50 | 12276,98 | 9569,78 | 12552,02 |
| 125,44 | 5,77 | 5,26 | 15 | 14 | 27756,24 | 21615,11 | 8634,44 | 6788,07 | 9315,96 |
| 177,31 | 5,53 | 5,97 | 16 | 15 | 33759,03 | 31136,81 | 10654,48 | 9595,27 | 11339,44 |
| 136,11 | 5,77 | 6,26 | 12 | 12 | 25780,45 | 23923,81 | 8537,71 | 7582,86 | 8797,62 |
| 116,23 | 4,81 | 4,30 | 17 | 16 | 26606,82 | 20539,09 | 9109,98 | 7378,50 | 8955,49 |
| 158,37 | 5,17 | 6,01 | 12 | 12 | 28540,82 | 28370,31 | 9358,89 | 8696,46 | 9595,45 |
| 151,77 | 5,35 | 5,18 | 15 | 14 | 31979,17 | 25967,92 | 10160,48 | 8304,26 | 10517,18 |
| 112,26 | 5,70 | 5,15 | 14 | 14 | 29957,13 | 27383,20 | 9950,34 | 8952,53 | 9971,77 |
| 120,32 | 6,36 | 5,31 | 15 | 13 | 30927,18 | 24863,16 | 9714,31 | 7790,43 | 10577,48 |
| 135,16 | 5,11 | 5,26 | 13 | 12 | 27455,26 | 24103,19 | 8955,96 | 7573,65 | 9144,62 |

| AUC_B_nV | AUC_C_V | AUC_C_nV | AUC_Exe_V | AUC_Exe_nV | AUC_Rec_V | AUC_Rec_nV |
|---|---|---|---|---|---|---|
| 7308,81 | 10095,58 | 7664,16 | 23245,04 | 16955,88 | 9565,90 | 8097,61 |
| 11154,96 | 13286,10 | 11160,61 | 29889,22 | 25445,18 | 12025,48 | 12221,53 |
| 10339,38 | 11870,27 | 10325,55 | 27086,73 | 24413,96 | 11103,57 | 10741,27 |
| 9293,87 | 10828,47 | 9765,20 | 23897,23 | 21857,87 | 9900,00 | 9317,93 |
| 7967,96 | 10398,45 | 8128,08 | 24524,80 | 18567,56 | 10039,69 | 8247,65 |
| 9130,18 | 12127,39 | 9253,77 | 25864,24 | 20278,16 | 12023,03 | 9848,79 |
| 7891,90 | 11159,94 | 8383,41 | 24676,27 | 18634,50 | 10489,33 | 8561,70 |
| 10306,33 | 13204,13 | 10710,07 | 29351,82 | 23429,37 | 12546,27 | 10671,09 |
| 6918,64 | 8646,48 | 6856,28 | 20550,96 | 15645,28 | 8439,45 | 7308,20 |
| 7305,58 | 10273,68 | 7192,90 | 23105,02 | 16384,72 | 9447,98 | 7985,47 |
| 7522,38 | 11566,66 | 7549,53 | 26427,37 | 17447,71 | 11055,04 | 7759,18 |
| 7075,72 | 10112,43 | 7227,86 | 23132,86 | 16418,33 | 8943,05 | 6948,24 |
| 9997,54 | 12828,96 | 10423,17 | 29825,11 | 22869,05 | 11751,23 | 11471,82 |
| 7281,91 | 9805,84 | 7545,13 | 21701,01 | 16831,63 | 9376,35 | 7888,23 |
| 10616,36 | 11765,12 | 10925,17 | 27062,17 | 24673,52 | 10266,08 | 10276,81 |
| 8062,96 | 8445,12 | 8277,99 | 20333,79 | 19087,41 | 8678,87 | 8200,18 |
| 6351,64 | 8541,34 | 6808,96 | 20901,40 | 16099,03 | 9059,73 | 7304,62 |
| 9619,61 | 9586,48 | 10054,24 | 22081,00 | 21789,55 | 9764,99 | 10470,21 |
| 8478,88 | 11301,51 | 9184,78 | 25097,57 | 20352,92 | 11199,24 | 9961,21 |
| 9235,67 | 10035,02 | 9195,00 | 23620,88 | 21764,05 | 9690,04 | 9298,12 |
| 8085,34 | 10635,39 | 8987,39 | 24459,69 | 19524,85 | 10320,01 | 8951,82 |
| 8241,43 | 9354,68 | 8288,12 | 21651,97 | 19127,02 | 9258,90 | 8475,41 |

| AUC_bas_V | AUC_bas_nV | AUC_Exe1_V | AUC_Exe1_nV | AUC_Rec1_V | AUC_Rec1_nV | AUC_Exe2_V |
|---|---|---|---|---|---|---|
| 1427,41 | 626,26 | 733,19 | 602,80 | 346,19 | 248,66 | 1075,12 |
| 1679,91 | 1610,50 | 824,73 | 854,97 | 396,20 | 410,20 | 1325,70 |
| 1498,39 | 2501,78 | 1097,11 | 1063,67 | 403,81 | 421,39 | 1596,90 |
| 890,54 | 1430,82 | 831,20 | 731,91 | 353,66 | 227,77 | 939,34 |
| 1106,43 | 1298,53 | 986,91 | 714,59 | 449,85 | 297,01 | 1334,90 |
| 876,39 | 1530,00 | 652,80 | 502,43 | 371,70 | 293,91 | 1246,19 |
| 1191,56 | 658,12 | 703,79 | 672,88 | 392,86 | 255,31 | 1054,82 |
| 2699,60 | 1976,84 | 949,25 | 672,42 | 491,82 | 434,31 | 1351,71 |
| 1378,64 | 1276,26 | 728,52 | 580,37 | 261,07 | 316,72 | 1025,63 |
| 2049,77 | 1170,79 | 764,15 | 643,01 | 371,52 | 279,68 | 1360,05 |
| 1904,53 | 2054,67 | 927,41 | 628,45 | 431,38 | 336,87 | 1171,81 |
| 1114,03 | 806,78 | 774,61 | 443,49 | 299,24 | 235,55 | 1200,45 |
| 1123,11 | 2430,40 | 1101,94 | 926,51 | 412,41 | 448,70 | 1636,25 |
| 1088,81 | 1056,48 | 722,01 | 614,91 | 332,38 | 231,62 | 1010,28 |
| 1655,77 | 1518,66 | 877,08 | 892,42 | 398,17 | 367,21 | 1297,09 |
| 1474,42 | 1420,70 | 793,98 | 556,25 | 353,75 | 227,89 | 1114,94 |
| 1188,49 | 1263,75 | 895,31 | 696,22 | 476,73 | 384,60 | 1090,64 |
| 1108,31 | 1045,34 | 1042,07 | 728,07 | 367,12 | 304,89 | 1387,35 |
| 1341,39 | 1317,41 | 757,69 | 720,82 | 311,67 | 399,62 | 1247,08 |
| 1264,85 | 1100,18 | 780,58 | 613,89 | 368,01 | 395,25 | 1536,24 |
| 1120,36 | 1282,03 | 821,85 | 588,81 | 420,93 | 287,35 | 1320,37 |
| 1184,83 | 22,94 | 715,74 | 486,44 | 353,62 | 281,85 | 1064,62 |

| AUC_Exe2_nV | AUC_Rec2_V | AUC_Rec2_nV | AUC_Exe3_V | AUC_Exe3_nV | AUC_Rec3_V |
|---|---|---|---|---|---|
| 872,87 | 334,84 | 284,84 | 1134,01 | 757,49 | 356,08 |
| 1200,01 | 526,42 | 517,70 | 1442,55 | 1186,70 | 405,40 |
| 1441,84 | 487,42 | 477,00 | 1387,89 | 1239,72 | 320,11 |
| 1180,61 | 413,58 | 340,37 | 1031,33 | 1078,79 | 289,93 |
| 997,06 | 406,59 | 313,09 | 1314,11 | 941,00 | 276,62 |
| 1039,71 | 508,25 | 386,97 | 1194,02 | 972,30 | 367,59 |
| 987,59 | 351,63 | 266,98 | 1297,11 | 1014,68 | 312,72 |
| 1014,32 | 586,51 | 471,34 | 1363,33 | 1052,94 | 370,63 |
| 718,27 | 324,67 | 359,38 | 991,57 | 710,23 | 312,70 |
| 953,39 | 192,64 | 315,40 | 1194,66 | 789,98 | 376,39 |
| 961,41 | 458,99 | 186,09 | 1406,75 | 975,19 | 432,95 |
| 979,69 | 260,74 | 297,09 | 1096,20 | 675,26 | 367,49 |
| 1274,17 | 620,26 | 465,72 | 1365,28 | 1037,68 | 439,56 |
| 754,92 | 428,56 | 255,14 | 1196,85 | 964,03 | 349,25 |
| 1037,30 | 343,65 | 371,83 | 1702,00 | 1301,03 | 348,37 |
| 1003,35 | 324,58 | 290,21 | 1189,06 | 1047,47 | 321,41 |
| 949,74 | 346,69 | 289,98 | 1054,17 | 981,47 | 290,14 |
| 931,40 | 499,55 | 394,71 | 1002,20 | 1285,78 | 332,42 |
| 955,13 | 500,82 | 324,60 | 1471,51 | 1059,68 | 306,78 |
| 1230,48 | 645,78 | 457,75 | 1189,26 | 1111,41 | 258,82 |
| 1044,81 | 372,65 | 334,67 | 1110,69 | 989,93 | 313,53 |
| 887,03 | 345,04 | 239,72 | 1198,25 | 1010,66 | 311,07 |

| AUC_Rec3_nV | AUC_Exe4_V | AUC_Exe4_nV | AUC_Rec4_V | AUC_Rec4_nV | AUC_Exe5_V |
|---|---|---|---|---|---|
| 258,81 | 1200,92 | 826,20 | 364,84 | 279,85 | 1535,40 |
| 401,43 | 1448,76 | 1291,43 | 546,86 | 507,69 | 2062,59 |
| 303,98 | 1475,64 | 1266,92 | 569,87 | 493,49 | 1645,94 |
| 266,88 | 1211,76 | 999,75 | 456,40 | 353,56 | 1395,10 |
| 206,40 | 1469,41 | 1007,95 | 449,16 | 418,03 | 1594,80 |
| 331,53 | 1277,34 | 959,18 | 442,99 | 422,81 | 1687,74 |
| 296,52 | 1181,39 | 955,58 | 460,74 | 404,78 | 1562,55 |
| 469,41 | 1561,80 | 1040,05 | 587,99 | 351,15 | 1940,63 |
| 222,61 | 1168,58 | 888,30 | 356,21 | 308,66 | 1471,81 |
| 203,72 | 1028,25 | 829,23 | 372,38 | 259,57 | 1503,59 |
| 260,08 | 1315,19 | 928,47 | 479,21 | 330,88 | 1510,80 |
| 285,09 | 1254,94 | 833,70 | 309,72 | 257,64 | 1497,63 |
| 428,37 | 1645,29 | 1217,71 | 535,69 | 485,21 | 1988,25 |
| 292,79 | 1056,24 | 871,54 | 368,87 | 288,02 | 1307,46 |
| 276,21 | 1264,46 | 1267,86 | 519,24 | 365,42 | 1861,29 |
| 283,54 | 1133,98 | 976,62 | 391,00 | 309,95 | 1363,02 |
| 257,72 | 1256,37 | 934,35 | 417,31 | 336,48 | 1485,79 |
| 332,44 | 1134,06 | 1032,49 | 467,64 | 438,37 | 1445,41 |
| 335,37 | 1368,98 | 1084,27 | 474,80 | 375,70 | 1573,84 |
| 264,30 | 1163,84 | 1431,50 | 470,48 | 354,18 | 1597,23 |
| 263,34 | 1210,85 | 961,11 | 303,80 | 357,33 | 1706,34 |
| 282,49 | 1221,51 | 1015,12 | 296,68 | 351,45 | 1387,51 |

| AUC_Exe5_nV | AUC_Rec5_V | AUC_Rec5_nV | AUC_Exe6_V | AUC_Exe6_nV | AUC_Rec6_V |
|---|---|---|---|---|---|
| 1198,62 | 465,00 | 430,64 | 1525,40 | 1136,33 | 1539,86 |
| 1703,47 | 551,71 | 640,48 | 1743,41 | 1720,14 | 1853,06 |
| 1575,65 | 624,08 | 595,87 | 1649,03 | 1611,07 | 1567,24 |
| 1360,88 | 486,25 | 430,34 | 1617,28 | 1384,62 | 1486,63 |
| 1223,17 | 502,23 | 359,30 | 1495,86 | 1162,62 | 1588,97 |
| 1442,69 | 615,90 | 467,18 | 1677,04 | 1359,64 | 1891,74 |
| 1210,28 | 530,96 | 424,23 | 1607,12 | 1092,55 | 1588,49 |
| 1554,45 | 653,78 | 561,06 | 1787,57 | 1606,07 | 1969,44 |
| 1104,46 | 445,10 | 325,57 | 1378,40 | 985,43 | 1377,30 |
| 1056,85 | 509,09 | 449,50 | 1436,55 | 1072,60 | 1385,04 |
| 1036,20 | 502,96 | 373,91 | 1597,14 | 1079,72 | 1877,22 |
| 1001,66 | 448,80 | 341,36 | 1420,30 | 1030,88 | 1476,87 |
| 1335,18 | 600,80 | 469,40 | 1931,25 | 1481,14 | 1772,57 |
| 1009,15 | 480,47 | 371,42 | 1382,08 | 1134,51 | 1505,54 |
| 1588,04 | 470,11 | 517,74 | 1573,01 | 1610,21 | 1498,98 |
| 1295,80 | 350,14 | 364,07 | 1201,85 | 1227,71 | 1506,65 |
| 1114,20 | 448,69 | 374,90 | 1348,13 | 1058,83 | 1462,37 |
| 1470,74 | 427,87 | 449,42 | 1253,20 | 1328,14 | 1456,69 |
| 1378,43 | 574,29 | 373,29 | 1573,01 | 1297,34 | 1849,43 |
| 1278,53 | 534,01 | 492,39 | 1406,09 | 1322,86 | 1444,11 |
| 1218,24 | 522,50 | 411,47 | 1610,80 | 1333,36 | 1786,13 |
| 1313,47 | 489,35 | 407,09 | 1572,55 | 1298,34 | 1450,87 |

| AUC_Rec6_nV | AUC_Exe7_V | AUC_Exe7_nV | AUC_Rec7_V | AUC_Rec7_nV | AUC_Exe8_V |
|---|---|---|---|---|---|
| 1433,38 | 992,64 | 730,44 | 466,82 | 334,95 | 1160,10 |
| 2138,22 | 1280,74 | 1128,41 | 498,04 | 425,90 | 1427,31 |
| 1664,04 | 1299,03 | 1134,14 | 459,40 | 393,92 | 1507,40 |
| 1590,24 | 1086,69 | 1029,50 | 380,29 | 339,97 | 1185,38 |
| 1379,99 | 1124,79 | 820,12 | 432,34 | 320,17 | 1413,62 |
| 1630,03 | 1004,33 | 877,64 | 529,59 | 362,55 | 1440,70 |
| 1411,79 | 1042,75 | 748,02 | 578,27 | 367,12 | 1176,45 |
| 1733,75 | 1342,25 | 1009,50 | 586,58 | 409,53 | 1571,45 |
| 1114,41 | 931,44 | 754,27 | 300,31 | 312,01 | 1063,62 |
| 1377,38 | 934,19 | 697,00 | 440,89 | 405,61 | 1453,63 |
| 1244,69 | 1087,58 | 727,64 | 475,14 | 374,00 | 1819,70 |
| 1210,20 | 1019,25 | 701,74 | 402,49 | 242,88 | 1229,16 |
| 1918,67 | 1363,07 | 951,25 | 506,41 | 511,37 | 1894,31 |
| 1344,39 | 897,81 | 711,31 | 333,47 | 240,67 | 1097,75 |
| 1663,68 | 1070,35 | 1107,37 | 498,40 | 447,40 | 1434,34 |
| 1474,58 | 948,64 | 852,00 | 434,62 | 230,84 | 1076,69 |
| 1278,83 | 1092,03 | 768,43 | 377,93 | 339,54 | 977,52 |
| 1564,01 | 981,73 | 824,77 | 473,32 | 411,30 | 1292,25 |
| 1798,44 | 1063,96 | 677,92 | 513,90 | 404,25 | 1422,48 |
| 1637,81 | 1144,41 | 852,26 | 413,43 | 326,11 | 1356,62 |
| 1676,14 | 1126,28 | 834,79 | 487,54 | 447,42 | 1559,72 |
| 1366,38 | 888,83 | 811,07 | 375,50 | 296,68 | 1017,09 |

| AUC_Exe8_nV | AUC_Rec8_V | AUC_Rec8_nV | AUC_Exe9_V | AUC_Exe9_nV | AUC_Rec9_V |
|---|---|---|---|---|---|
| 839,01 | 489,72 | 308,94 | 1244,84 | 799,93 | 339,65 |
| 1329,17 | 564,22 | 410,00 | 1640,42 | 1431,90 | 326,76 |
| 1366,10 | 467,50 | 408,85 | 1235,00 | 1237,86 | 351,84 |
| 1224,27 | 380,75 | 388,38 | 1206,76 | 1163,12 | 312,52 |
| 1073,16 | 466,77 | 345,70 | 1245,68 | 923,54 | 359,99 |
| 1135,31 | 537,83 | 588,81 | 1352,96 | 1068,60 | 368,77 |
| 1005,99 | 443,67 | 267,19 | 1363,04 | 1073,70 | 400,23 |
| 1312,82 | 567,72 | 520,97 | 1402,31 | 1096,90 | 351,35 |
| 1010,28 | 327,72 | 269,68 | 1065,47 | 830,55 | 298,64 |
| 924,73 | 420,12 | 286,93 | 1180,42 | 868,59 | 313,31 |
| 878,57 | 406,64 | 267,28 | 1583,11 | 1055,21 | 423,43 |
| 977,01 | 341,75 | 188,97 | 1231,02 | 870,76 | 327,14 |
| 1452,75 | 627,38 | 351,75 | 1567,12 | 1206,60 | 490,56 |
| 903,11 | 477,08 | 311,99 | 1264,88 | 896,51 | 333,22 |
| 1187,52 | 496,17 | 523,19 | 1467,22 | 1392,42 | 346,13 |
| 1045,27 | 438,29 | 300,05 | 1139,23 | 1110,69 | 317,28 |
| 725,84 | 459,45 | 262,49 | 1058,89 | 799,82 | 207,22 |
| 1399,64 | 509,13 | 568,89 | 1071,31 | 1195,10 | 305,58 |
| 959,97 | 337,20 | 343,89 | 1352,88 | 1116,96 | 314,07 |
| 1430,98 | 479,48 | 567,42 | 1111,03 | 1164,61 | 263,12 |
| 1085,22 | 454,32 | 301,77 | 1158,00 | 998,34 | 314,82 |
| 908,49 | 319,81 | 400,32 | 1165,15 | 1049,57 | 359,33 |

| AUC_Rec9_nV | AUC_Exe10_V | AUC_Exe10_nV | AUC_Rec10_V | AUC_Rec10_nV | AUC_Exe11_V |
|---|---|---|---|---|---|
| 258,23 | 1320,19 | 885,46 | 459,46 | 361,37 | 1628,42 |
| 350,50 | 1685,63 | 1360,93 | 538,69 | 511,86 | 2329,66 |
| 329,20 | 1446,45 | 1317,55 | 588,75 | 438,24 | 1637,55 |
| 283,23 | 1403,21 | 1137,68 | 455,54 | 462,25 | 1602,48 |
| 338,93 | 1339,21 | 1029,00 | 478,39 | 202,67 | 1554,23 |
| 369,50 | 1382,21 | 1003,44 | 478,66 | 425,53 | 1843,74 |
| 330,39 | 1401,90 | 929,23 | 441,74 | 364,31 | 1745,86 |
| 282,38 | 1766,96 | 1409,61 | 570,63 | 483,03 | 2017,50 |
| 259,75 | 1190,07 | 860,28 | 363,50 | 294,00 | 1402,32 |
| 262,75 | 1145,69 | 880,58 | 416,20 | 303,61 | 1606,13 |
| 302,76 | 1457,84 | 995,54 | 417,63 | 389,14 | 1722,04 |
| 260,79 | 1304,51 | 929,47 | 447,75 | 309,28 | 1470,47 |
| 412,42 | 1658,85 | 1343,19 | 469,51 | 496,93 | 1695,20 |
| 287,35 | 1246,05 | 984,86 | 434,08 | 345,14 | 1441,58 |
| 366,79 | 1369,65 | 1378,81 | 476,14 | 414,50 | 1791,64 |
| 279,03 | 1160,02 | 1066,46 | 443,45 | 317,50 | 1298,13 |
| 146,09 | 1201,98 | 781,77 | 411,39 | 239,53 | 1357,74 |
| 364,49 | 1266,16 | 1155,34 | 444,44 | 467,45 | 1424,96 |
| 294,63 | 1394,94 | 1141,24 | 494,20 | 467,88 | 1666,64 |
| 236,60 | 1332,85 | 1070,49 | 462,37 | 351,02 | 1456,47 |
| 246,62 | 1238,62 | 866,95 | 519,81 | 368,62 | 1621,30 |
| 371,81 | 1183,10 | 932,94 | 448,93 | 274,18 | 1428,93 |

| AUC_Exe11_nV | AUC_Rec11_V | AUC_Rec11_nV | AUC_Exe12_V | AUC_Exe12_nV | AUC_Rec12_V |
|---|---|---|---|---|---|
| 1208,94 | 412,71 | 456,26 | 1537,72 | 1125,27 | 1659,65 |
| 1799,30 | 448,53 | 671,28 | 1977,38 | 1735,72 | 2124,41 |
| 1550,44 | 571,77 | 606,48 | 1739,93 | 1556,61 | 1650,15 |
| 1348,40 | 498,88 | 392,41 | 1707,05 | 1524,65 | 1627,04 |
| 1282,81 | 514,74 | 563,80 | 1447,72 | 1068,07 | 1497,63 |
| 1472,63 | 606,08 | 455,63 | 1668,78 | 1370,54 | 2143,45 |
| 1265,86 | 545,48 | 368,99 | 1628,60 | 1171,09 | 1601,87 |
| 1601,40 | 547,51 | 474,09 | 1915,89 | 1706,11 | 1914,09 |
| 1073,75 | 414,00 | 340,92 | 1366,87 | 913,15 | 1390,38 |
| 1163,97 | 558,91 | 480,27 | 1495,31 | 1031,54 | 1367,60 |
| 1023,35 | 519,52 | 360,52 | 1627,35 | 1148,36 | 1712,25 |
| 1060,79 | 403,66 | 360,43 | 1450,79 | 1173,58 | 1499,23 |
| 1379,31 | 604,07 | 458,21 | 1675,55 | 1433,74 | 1662,00 |
| 1105,80 | 444,21 | 402,35 | 1345,82 | 1092,80 | 1402,16 |
| 1664,11 | 641,29 | 555,96 | 1748,10 | 1578,30 | 1683,63 |
| 1280,86 | 397,42 | 424,02 | 1143,86 | 1156,25 | 1409,44 |
| 906,51 | 453,00 | 326,41 | 1358,33 | 1055,21 | 1485,85 |
| 1492,21 | 432,99 | 409,43 | 1393,59 | 1330,99 | 1428,02 |
| 1337,38 | 485,43 | 501,35 | 1471,48 | 1233,41 | 1901,12 |
| 1484,29 | 514,95 | 417,00 | 1437,03 | 1334,88 | 1438,51 |
| 1324,01 | 473,27 | 394,74 | 1623,81 | 1216,86 | 1641,30 |
| 1445,59 | 474,35 | 406,04 | 1483,60 | 1344,73 | 1451,04 |

| AUC_Rec12_nV | AUC_Exe13_V | AUC_Exe13_nV | AUC_Rec13_V | AUC_Rec13_nV | AUC_Exe14_V |
|---|---|---|---|---|---|
| 1446,01 | 1153,99 | 795,22 | 375,20 | 349,72 | 1326,66 |
| 2239,19 | 1422,46 | 1147,06 | 476,31 | 443,00 | 1508,42 |
| 1853,72 | 1209,84 | 1041,55 | 484,24 | 444,24 | 1630,70 |
| 1696,57 | 1149,71 | 1056,23 | 437,24 | 396,58 | 1280,98 |
| 1436,27 | 1169,32 | 811,80 | 456,19 | 373,04 | 1355,65 |
| 1614,94 | 1112,20 | 892,79 | 507,55 | 371,51 | 1486,60 |
| 1499,58 | 1148,27 | 800,99 | 535,21 | 371,17 | 1234,41 |
| 1713,64 | 1342,46 | 1002,61 | 647,53 | 541,10 | 1619,97 |
| 1200,06 | 909,54 | 655,80 | 393,14 | 305,84 | 1034,82 |
| 1265,22 | 985,35 | 741,20 | 446,05 | 306,35 | 1429,06 |
| 1405,56 | 1192,39 | 681,27 | 504,90 | 370,01 | 1634,20 |
| 1171,94 | 1083,81 | 715,80 | 471,69 | 283,60 | 1325,29 |
| 1876,00 | 1315,30 | 1061,74 | 432,32 | 516,43 | 1918,33 |
| 1384,44 | 999,79 | 744,20 | 354,84 | 345,59 | 1251,12 |
| 1658,10 | 1216,38 | 1105,06 | 444,99 | 435,25 | 2062,25 |
| 1579,15 | 957,04 | 845,98 | 296,72 | 339,61 | 1211,68 |
| 1269,66 | 1104,14 | 796,95 | 364,06 | 301,29 | 1028,48 |
| 1721,02 | 1001,33 | 835,15 | 455,43 | 452,29 | 1252,76 |
| 1761,41 | 1094,87 | 868,69 | 668,31 | 338,04 | 1393,07 |
| 1586,52 | 1212,78 | 959,53 | 388,55 | 320,26 | 1377,71 |
| 1524,42 | 1175,66 | 892,78 | 533,89 | 356,26 | 1394,43 |
| 1508,25 | 935,48 | 751,05 | 339,48 | 299,21 | 1017,89 |

| AUC_Exe14_nV | AUC_Rec14_V | AUC_Rec14_nV | AUC_Exe15_V | AUC_Exe15_nV | AUC_Rec15_V |
|---|---|---|---|---|---|
| 886,80 | 373,43 | 290,33 | 1210,22 | 810,71 | 304,77 |
| 1279,22 | 544,58 | 504,61 | 1688,68 | 1412,97 | 378,84 |
| 1377,42 | 526,78 | 383,01 | 1345,27 | 1284,44 | 318,58 |
| 1179,54 | 402,65 | 403,47 | 1274,29 | 1268,73 | 365,52 |
| 1060,78 | 454,90 | 310,50 | 1233,44 | 1022,52 | 330,26 |
| 1060,76 | 586,24 | 488,37 | 1532,65 | 1301,49 | 388,59 |
| 957,62 | 455,70 | 342,84 | 1408,74 | 1125,72 | 327,26 |
| 1222,36 | 612,70 | 447,47 | 1540,01 | 1264,62 | 403,62 |
| 878,70 | 406,49 | 374,55 | 1014,65 | 836,55 | 263,75 |
| 945,20 | 433,48 | 323,37 | 1109,13 | 851,63 | 363,02 |
| 1006,91 | 468,86 | 229,93 | 1548,86 | 1117,25 | 397,69 |
| 895,91 | 344,12 | 226,78 | 1270,98 | 954,01 | 301,08 |
| 1304,01 | 619,89 | 612,10 | 1589,06 | 1241,40 | 406,94 |
| 846,93 | 415,30 | 362,72 | 1275,45 | 979,80 | 366,27 |
| 1306,33 | 452,79 | 534,65 | 1325,67 | 1362,25 | 414,57 |
| 1091,52 | 361,00 | 417,58 | 1113,96 | 1010,74 | 252,90 |
| 759,01 | 471,80 | 295,44 | 986,01 | 794,91 | 214,31 |
| 1395,79 | 517,48 | 635,08 | 1097,89 | 1232,03 | 304,12 |
| 1308,20 | 348,64 | 343,33 | 1464,17 | 1131,60 | 399,04 |
| 1638,38 | 393,89 | 435,98 | 1233,47 | 1091,21 | 220,72 |
| 1151,54 | 437,55 | 430,74 | 1192,41 | 1089,64 | 333,78 |
| 913,38 | 349,89 | 309,66 | 1199,73 | 1117,45 | 368,92 |

| AUC_Rec15_nV | AUC_Exe16_V | AUC_Exe16_nV | AUC_Rec16_V | AUC_Rec16_nV | AUC_Exe17_V |
|---|---|---|---|---|---|
| 299,60 | 1339,36 | 974,65 | 441,93 | 333,13 | 1573,80 |
| 346,79 | 1774,55 | 1320,12 | 541,84 | 578,69 | 2250,71 |
| 307,80 | 1521,03 | 1347,35 | 548,31 | 541,41 | 1817,72 |
| 330,21 | 1630,16 | 1291,39 | 447,83 | 460,56 | 1620,66 |
| 277,90 | 1372,30 | 1071,40 | 512,01 | 443,69 | 1582,62 |
| 471,62 | 1499,38 | 1350,25 | 570,09 | 482,51 | 1945,33 |
| 268,17 | 1494,13 | 1105,95 | 507,59 | 445,85 | 1392,18 |
| 341,47 | 1797,02 | 1404,45 | 576,69 | 470,23 | 2034,91 |
| 250,35 | 1231,31 | 882,45 | 450,84 | 316,56 | 1254,11 |
| 277,46 | 1241,70 | 861,46 | 476,96 | 432,06 | 1637,21 |
| 269,67 | 1444,67 | 972,13 | 457,21 | 361,48 | 1673,76 |
| 323,31 | 1412,17 | 947,51 | 381,84 | 259,40 | 1606,30 |
| 501,33 | 1752,22 | 1358,01 | 501,09 | 454,77 | 1851,02 |
| 294,34 | 1266,23 | 1000,73 | 434,33 | 354,88 | 1480,60 |
| 359,84 | 1436,95 | 1472,92 | 392,93 | 424,89 | 1821,09 |
| 278,34 | 1102,46 | 1022,29 | 426,79 | 394,05 | 1206,80 |
| 239,02 | 1076,94 | 909,01 | 326,97 | 325,75 | 1341,52 |
| 296,76 | 1223,81 | 1263,79 | 489,99 | 505,93 | 1488,49 |
| 307,86 | 1461,60 | 1207,50 | 575,51 | 397,73 | 1700,96 |
| 260,59 | 1343,90 | 1000,95 | 457,76 | 321,78 | 1515,14 |
| 258,66 | 1268,99 | 984,57 | 473,79 | 398,67 | 1672,11 |
| 249,22 | 1263,71 | 1222,73 | 473,76 | 372,57 | 1442,49 |

| AUC_Exe17_nV | AUC_Rec17_V | AUC_Rec17_nV | AUC_Exe18_V | AUC_Exe18_nV | AUC_Rec18_V |
|---|---|---|---|---|---|
| 1231,40 | 443,15 | 418,87 | 1553,07 | 1273,72 | 392,58 |
| 1852,74 | 644,22 | 584,47 | 2055,51 | 1690,93 | 659,40 |
| 1439,92 | 623,49 | 596,70 | 1844,30 | 1561,72 | 540,25 |
| 1441,86 | 495,60 | 480,68 | 1723,84 | 1455,95 | 609,70 |
| 1230,33 | 441,54 | 398,49 | 1490,23 | 1127,62 | 421,52 |
| 1489,39 | 638,52 | 365,70 | 1860,24 | 979,37 | 469,49 |
| 1302,24 | 423,29 | 448,34 | 2233,18 | 1214,52 | 591,60 |
| 1687,93 | 582,42 | 557,03 | 2046,81 | 1770,79 | 525,26 |
| 1071,99 | 365,63 | 393,04 | 1322,21 | 890,45 | 388,01 |
| 966,65 | 551,77 | 380,39 | 1599,96 | 1107,13 | 452,59 |
| 1109,30 | 537,36 | 308,83 | 1706,77 | 1122,76 | 551,71 |
| 1082,36 | 430,19 | 394,48 | 1484,98 | 1144,70 | 429,28 |
| 1501,65 | 565,96 | 508,73 | 1876,82 | 1363,00 | 483,82 |
| 1079,70 | 502,90 | 399,44 | 1459,00 | 1136,78 | 413,43 |
| 1700,02 | 453,89 | 502,42 | 1743,59 | 1721,55 | 386,61 |
| 1293,66 | 337,31 | 379,71 | 1178,45 | 1204,50 | 316,12 |
| 1021,99 | 439,71 | 320,83 | 1187,39 | 1044,77 | 406,09 |
| 1524,55 | 432,76 | 549,31 | 1322,43 | 1363,56 | 420,45 |
| 1539,89 | 576,95 | 407,46 | 1618,40 | 1334,48 | 567,09 |
| 1378,06 | 464,87 | 418,53 | 1426,21 | 1369,74 | 471,17 |
| 1533,84 | 505,31 | 480,64 | 1647,47 | 1410,03 | 425,10 |
| 1288,37 | 497,56 | 433,88 | 1465,78 | 1330,61 | 553,70 |

| AUC_Rec18_nV | DiffAUC_RecExe_V | DiffAUC_RecExe_nV |
|---|---|---|
| 304,03 | 83,38 | 70,71 |
| 539,52 | 85,24 | 70,21 |
| 481,93 | 83,70 | 77,78 |
| 474,46 | 82,83 | 80,45 |
| 262,69 | 83,81 | 76,97 |
| 319,67 | 73,07 | 69,24 |
| 428,14 | 80,69 | 74,08 |
| 409,13 | 80,22 | 74,83 |
| 344,10 | 83,56 | 72,64 |
| 376,19 | 83,91 | 68,93 |
| 387,48 | 82,02 | 76,87 |
| 299,44 | 88,48 | 81,06 |
| 555,69 | 86,94 | 66,38 |
| 375,93 | 79,32 | 72,36 |
| 491,74 | 89,99 | 82,38 |
| 310,06 | 80,34 | 79,80 |
| 316,07 | 79,05 | 75,15 |
| 604,42 | 77,35 | 70,18 |
| 786,36 | 76,58 | 68,56 |
| 454,64 | 83,64 | 80,26 |
| 412,96 | 81,31 | 74,26 |
| 624,60 | 80,19 | 77,18 |
