# Supplementary material for: Metabolic effect of bodyweight whole-body vibration in a 20-min exercise session: A crossover study using verified vibration stimulus
Source: PLoS One. 2018 Jan 31;13(1):e0192046. doi: 10.1371/journal.pone.0192046 (PMC5792008; doi:10.1371/journal.pone.0192046)
Supplement: S2 File — (PDF) [file pone.0192046.s002.pdf]

Variable name	Description<br>V = vibration; nV = no Vibration
Id	Subject ID
Age	Age of participants (years)
Sequence	Sequence of treatments (nV_V/V_nV)
PeakAccel	Peak acceleration (g)
BodyMass	Body mass (Kg)
Height	Stature (cm)
BMI	BMI (kg/m^2)
HR_bas	Mean basal heart rate (bpm)
HR_V	Mean heart rate (bpm) - Vibration
HR_nV	Mean heart rate (bpm) - no Vibration
EE_V	EE tot (Kcal) - Vibration
EE_nV	EE tot (Kcal) - no Vibration
METs_V	METs Tot - Vibration
METs_nV	METs Tot - no Vibration
RPE_V	RPE - Vibration
RPE_nV	RPE - no Vibration
AUC_V	O2 AUC (mL) - Tot - Vibration
AUC_nV	O2 AUC (mL) - Tot - no Vibration
AUC_A_V	O2 AUC (mL) - Set A - Vibration
AUC_A_nV	O2 AUC (mL) - Set A - no Vibration
AUC_B_V	O2 AUC (mL) - Set B - Vibration
AUC_B_nV	O2 AUC (mL) - Set B - no Vibration
AUC_C_V	O2 AUC (mL) - Set C - Vibration
AUC_C_nV	O2 AUC (mL) - Set C - no Vibration
AUC_Exe_V	O2 AUC (mL) - Tot exercise time - Vibration
AUC_Exe_nV	O2 AUC (mL) - Tot exercise time - no Vibration
AUC_Rec_V	O2 AUC (mL) - Tot recovery time - Vibration
AUC_Rec_nV	O2 AUC (mL) - Tot recovery time - no Vibration
AUC_bas_V	O2 AUC (ml) - Baseline - Vibration
AUC_bas_nV	O2 AUC (ml) - Baseline - no Vibration
AUC_Exe1_V	O2 AUC (ml) - Exercise 1 - Vibration
AUC_Exe1_nV	O2 AUC (ml) - Exercise 1 - no Vibration
AUC_Rec1_V	O2 AUC (ml) - Recovery 1 - Vibration
AUC_Rec1_nV	O2 AUC (ml) - Recovery 1 - no Vibration
AUC_Exe2_V	O2 AUC (ml) - Exercise 2 - Vibration
AUC_Exe2_nV	O2 AUC (ml) - Exercise 2 - no Vibration
AUC_Rec2_V	O2 AUC (ml) - Recovery 2 - Vibration
AUC_Rec2_nV	O2 AUC (ml) - Recovery 2 - no Vibration
AUC_Exe3_V	O2 AUC (ml) - Exercise 3 - Vibration
AUC_Exe3_nV	O2 AUC (ml) - Exercise 3 - no Vibration
AUC_Rec3_V	O2 AUC (ml) - Recovery 3 - Vibration
AUC_Rec3_nV	O2 AUC (ml) - Recovery 3 - no Vibration
AUC_Exe4_V	O2 AUC (ml) - Exercise 4 - Vibration
AUC_Exe4_nV	O2 AUC (ml) - Exercise 4 - no Vibration
AUC_Rec4_V	O2 AUC (ml) - Recovery 4 - Vibration
AUC_Rec4_nV	O2 AUC (ml) - Recovery 4 - no Vibration
AUC_Exe5_V	O2 AUC (ml) - Exercise 5 - Vibration
AUC_Exe5_nV	O2 AUC (ml) - Exercise 5 - no Vibration
AUC_Rec5_V	O2 AUC (ml) - Recovery 5 - Vibration
AUC_Rec5_nV	O2 AUC (ml) - Recovery 5 - no Vibration
AUC_Exe6_V	O2 AUC (ml) - Exercise 6 - Vibration
AUC_Exe6_nV	O2 AUC (ml) - Exercise 6 - no Vibration
AUC_Rec6_V	O2 AUC (ml) - Recovery 6 - Vibration
AUC_Rec6_nV	O2 AUC (ml) - Recovery 6 - no Vibration

AUC_Exe7_V	O2 AUC (ml) - Exercise 7 - Vibration
AUC_Exe7_nV	O2 AUC (ml) - Exercise 7 - no Vibration
AUC_Rec7_V	O2 AUC (ml) - Recovery 7 - Vibration
AUC_Rec7_nV	O2 AUC (ml) - Recovery 7 - no Vibration
AUC_Exe8_V	O2 AUC (ml) - Exercise 8 - Vibration
AUC_Exe8_nV	O2 AUC (ml) - Exercise 8 - no Vibration
AUC_Rec8_V	O2 AUC (ml) - Recovery 8 - Vibration
AUC_Rec8_nV	O2 AUC (ml) - Recovery 8 - no Vibration
AUC_Exe9_V	O2 AUC (ml) - Exercise 9 - Vibration
AUC_Exe9_nV	O2 AUC (ml) - Exercise 9 - no Vibration
AUC_Rec9_V	O2 AUC (ml) - Recovery 9 - Vibration
AUC_Rec9_nV	O2 AUC (ml) - Recovery 9 - no Vibration
AUC_Exe10_V	O2 AUC (ml) - Exercise 10 - Vibration
AUC_Exe10_nV	O2 AUC (ml) - Exercise 10 - no Vibration
AUC_Rec10_V	O2 AUC (ml) - Recovery 10 - Vibration
AUC_Rec10_nV	O2 AUC (ml) - Recovery 10 - no Vibration
AUC_Exe11_V	O2 AUC (ml) - Exercise 11 - Vibration
AUC_Exe11_nV	O2 AUC (ml) - Exercise 11 - no Vibration
AUC_Rec11_V	O2 AUC (ml) - Recovery 11 - Vibration
AUC_Rec11_nV	O2 AUC (ml) - Recovery 11 - no Vibration
AUC_Exe12_V	O2 AUC (ml) - Exercise 12 - Vibration
AUC_Exe12_nV	O2 AUC (ml) - Exercise 12 - no Vibration
AUC_Rec12_V	O2 AUC (ml) - Recovery 12 - Vibration
AUC_Rec12_nV	O2 AUC (ml) - Recovery 12 - no Vibration
AUC_Exe13_V	O2 AUC (ml) - Exercise 13 - Vibration
AUC_Exe13_nV	O2 AUC (ml) - Exercise 13 - no Vibration
AUC_Rec13_V	O2 AUC (ml) - Recovery 13 - Vibration
AUC_Rec13_nV	O2 AUC (ml) - Recovery 13 - no Vibration
AUC_Exe14_V	O2 AUC (ml) - Exercise 14 - Vibration
AUC_Exe14_nV	O2 AUC (ml) - Exercise 14 - no Vibration
AUC_Rec14_V	O2 AUC (ml) - Recovery 14 - Vibration
AUC_Rec14_nV	O2 AUC (ml) - Recovery 14 - no Vibration
AUC_Exe15_V	O2 AUC (ml) - Exercise 15 - Vibration
AUC_Exe15_nV	O2 AUC (ml) - Exercise 15 - no Vibration
AUC_Rec15_V	O2 AUC (ml) - Recovery 15 - Vibration
AUC_Rec15_nV	O2 AUC (ml) - Recovery 15 - no Vibration
AUC_Exe16_V	O2 AUC (ml) - Exercise 16 - Vibration
AUC_Exe16_nV	O2 AUC (ml) - Exercise 16 - no Vibration
AUC_Rec16_V	O2 AUC (ml) - Recovery 16 - Vibration
AUC_Rec16_nV	O2 AUC (ml) - Recovery 16 - no Vibration
AUC_Exe17_V	O2 AUC (ml) - Exercise 17 - Vibration
AUC_Exe17_nV	O2 AUC (ml) - Exercise 17 - no Vibration
AUC_Rec17_V	O2 AUC (ml) - Recovery 17 - Vibration
AUC_Rec17_nV	O2 AUC (ml) - Recovery 17 - no Vibration
AUC_Exe18_V	O2 AUC (ml) - Exercise 18 - Vibration
AUC_Exe18_nV	O2 AUC (ml) - Exercise 18 - no Vibration
AUC_Rec18_V	O2 AUC (ml) - Recovery 18 - Vibration
AUC_Rec18_nV	O2 AUC (ml) - Recovery 18 - no Vibration
DiffAUC_RecExe_V	O2(AUC) % decrease betw. exercise and recovery period - Vibration
DiffAUC_RecExe_nV	O2(AUC) % decrease betw. exercise and recovery period - no Vibration
